# Development and validation of the AI-empowered visualization teaching competency scale

**DOI:** 10.3389/fpsyg.2026.1994352

**Published:** 2026-10-21

**Authors:** Longlong Chen, Shoujun Qiao, Xinbing Luo, Gang Chen, Yankui Zhang

**Affiliations:** 1 School of Teacher Development, Shaanxi Normal University, Xi’an, China; 2 Faculty of Education, Shaanxi Normal University, Xi’an, China; 3 School of Mathematics and Statistics, Shaanxi Normal University, Xi’an, China

**Keywords:** AI-empowered teaching, artificial intelligence, scale development and validation, teacher competency, visualization teaching

## Abstract

This study took primary and secondary school teachers in western China as participants. Grounded in the AI-TPACK framework and integrating visual literacy, data literacy, and relevant learning and development theories, it constructed a five-dimensional theoretical framework for teacher visualization teaching competency under AI empowerment. Drawing on item analysis, exploratory factor analysis, confirmatory factor analysis, reliability testing, measurement invariance testing, and criterion-related validity testing, the study developed and validated the AI-Empowered Teacher Visualization Teaching Competency Scale (AI-VTCS). The results show that AI-VTCS comprises five dimensions—Design and Creation, Awareness and Interpretation, Decision-Making and Collaboration, Ethics and Safety, and Professional Development—with a total of 31 items. The Cronbach’s α coefficients of the five subscales ranged from 0.911 to 0.934, and that of the overall scale was 0.967. Both the first-order and second-order five-factor models achieved good fit, and the moderate-to-high correlations among the five dimensions support the hierarchical nature of the construct. Measurement invariance from configural to strict invariance was established across gender, school type (public/private), and teaching stage (primary, middle, and high school). Convergent validity, discriminant validity, and criterion-related validity all met psychometric standards. This study not only fills the theoretical gap in existing teacher competency frameworks regarding AI-empowered visualization teaching, but also provides a reliable and valid standardized measurement instrument for teacher education institutions to assess and precisely enhance teachers’ AI visualization teaching competency.

## 1 Introduction

Artificial intelligence is permeating education and reshaping the fundamental structure of teaching and learning. With the rapid iteration of generative AI in text generation, image creation, and data visualization, the ways in which knowledge is represented in instruction have undergone a fundamental transformation: teachers are no longer merely consumers of visualized instructional resources, but are increasingly acting as decision-makers who employ AI tools to engage in visual design, generation, and critical evaluation. This shift imposes new demands on teacher competency, yet existing teacher competency frameworks have not yet adequately responded to it.

Over the past two decades, research on teacher technology integration has long been dominated by the TPACK framework (Mishra and Koehler, 2006; Khine et al., 2016), which conceptualizes technology integration competency as the product of the interplay among technological knowledge, pedagogical knowledge, and content knowledge. The technological underpinnings of TPACK, however, are rooted in the Web 2.0 era, centered on interactive whiteboards, multimedia courseware, and online learning platforms; it could not have anticipated the profound reshaping of instructional practice brought about by generative AI and large language models (Eutsler, 2021). In recent years, researchers have begun to develop dedicated frameworks for teacher AI competency. Among these, the Teacher AI Competency Self-Efficacy Scale (TAICS) developed by Chiu et al. (2024) has established a relatively comprehensive measurement system encompassing six dimensions: AI knowledge, AI pedagogy, AI assessment, AI ethics, humanistic education, and professional engagement. Nevertheless, neither TPACK nor TAICS conceptualizes visualization teaching competency as a standalone dimension. A scoping review by Sperling et al. (2024) further indicates that the field of teacher AI literacy remains dominated by theoretical discussions and qualitative research; validated instruments subjected to rigorous psychometric examination are already scarce, and measurement tools targeting the emergent dimension of visualization teaching competency are entirely absent.

Furthermore, terms such as visualization teaching competency and visual instructional design have often been used interchangeably in the literature, resulting in blurred conceptual boundaries—yet a clear construct is a prerequisite for scale development. Accordingly, this study delineates four interrelated yet distinct constructs. Visualization teaching competency is the broad, overarching construct, referring to teachers’ integrated competence in employing visual representations—such as static charts, diagrams, concept maps, animations, and data visualization—to design, implement, and evaluate instruction (Vázquez-Ingelmo et al., 2024). Visual instructional design is a nested design-oriented sub-competency, namely the ability to select appropriate visual representations according to the type of knowledge, instructional objectives, and learner characteristics, grounded in the cognitive principles of multimedia learning (Mayer, 2009; Sweller, 1988). AI knowledge refers to teachers’ understanding of AI technology itself, encompassing its generative logic, the boundaries of its tool capabilities, prompt design, and output evaluation, and is distinguished from pedagogical and content knowledge (Chiu et al., 2024; Hava and Babayiğit, 2024). Building on these three, this study defines AI-Empowered Visualization Teaching Competency (AI-VTC) as teachers’ integrated ability, in AI-empowered contexts, to fuse AI knowledge with the principles of visual instructional design and, through AI tools, to visually transform instructional content, generate resources, and critically evaluate them. Although generative AI has substantially lowered the threshold for creating instructional resources, it has simultaneously raised the demand for this competency from “operating tools” to “design thinking and critical evaluation” (Vázquez-Ingelmo et al., 2024), such that it can no longer be subsumed under the generic technology dimension of existing frameworks. Moreover, the mediating role of AI self-efficacy between AI attitudes and AI-TPACK (Mustafa et al., 2025; Dong et al., 2024) suggests that self-efficacy should be incorporated into the theoretical framework in order to fully capture the psychological mechanisms underlying this competency.

Accordingly, the present study aims to develop a valid and reliable scale for measuring teachers’ AI-Empowered Visualization Teaching Competency—that is, to establish valid and reliable dimensions and items constituting the AI-Empowered Visualization Teaching Competency Scale (AI-VTCS).

## 2 Theoretical framework

This study takes AI-TPACK as its core theoretical foundation and, drawing on related research on visualization teaching, visual instructional design, AI knowledge, and teacher self-efficacy, collectively delineates the connotation of AI-VTC.

### 2.1 TPACK and AI-TPACK

TPACK was proposed by Mishra and Koehler (2006) on the basis of pedagogical content knowledge (PCK). It conceptualizes the knowledge required for teachers to integrate technology effectively as seven components arising from the overlap among technological knowledge (TK), pedagogical knowledge (PK), and content knowledge (CK), with TPACK—the intersection of all three—occupying the central position. Its central claim is that teachers’ technology integration competence derives from the interplay of the three knowledge bases rather than from their simple aggregation (Khine et al., 2016).

Generative AI has placed new demands on the “technology” side of this framework. Within classical TPACK, technology consists of established tools that teachers operate proficiently; in AI-empowered classrooms, by contrast, technology itself can automatically generate texts, images, and visualization solutions in response to prompts. The teacher’s role thus shifts from “operating tools” to “co-creating with AI,” and the required technological knowledge accordingly expands to encompass understanding AI’s generative logic and capability boundaries, designing effective prompts, and evaluating the reliability and instructional appropriateness of AI outputs. AI-TPACK is precisely the theoretical extension of TPACK in response to this shift (Hava and Babayiğit, 2024).

### 2.2 Visualization teaching competency

Visualization teaching competency refers to teachers’ integrated competence in employing visual representations to design, implement, and evaluate instruction. Its intellectual roots lie in visual literacy, which Debes (1969) defined as an individual’s ability to understand, interpret, and evaluate visual information. The rise of generative AI and learning analytics has extended visual literacy from “interpreting given visual information” to “scrutinizing the generative mechanisms and credibility of information produced by AI” (Vázquez-Ingelmo et al., 2024). The instructional data literacy framework proposed by Gummer and Mandinach (2015) further emphasizes teachers’ ability to transform various forms of data into actionable instructional decisions. Accordingly, whereas visualization teaching competency has traditionally referred to the use of static representational tools such as charts and concept maps, in the AI era it extends toward critical interpretation and pedagogical reconfiguration.

### 2.3 Visual instructional design

Research on visual instructional design examines how teachers transform abstract disciplinary content into appropriate visual representations and organize instructional presentation accordingly. Its underlying premise is that visual presentation does not inherently promote learning: ill-chosen chart forms, redundant visual elements, or presentations mismatched to learners’ cognitive levels can instead extraneously increase cognitive load and impede understanding (Mayer, 2009). Prior research has further distinguished two related literacies—visualization literacy, which emphasizes “production,” and visual literacy, which emphasizes “interpreting and evaluating visual representations” (Schüller et al., 2019)—implying that visual instructional design concerns both the generation of representations and whether those representations can be correctly interpreted by students. Accordingly, the core issues in this field include selecting appropriate representational forms according to knowledge types and instructional objectives (e.g., structural diagrams, flowcharts, statistical graphs, or animations), adjusting presentation pace and detail density in light of learner characteristics, and organizing discrete visual materials into coherent instructional sequences so that visual representations serve conceptual understanding rather than functioning merely as decoration. Its design principles are grounded in research on multimedia learning and cognitive load (Mayer, 2009; Sweller, 1988).

### 2.4 AI knowledge

AI knowledge refers to teachers’ understanding of AI technology itself, including its generative logic, the boundaries of its tool capabilities, prompt design, and output evaluation, and is distinguished from pedagogical knowledge and content knowledge. Chiu et al. (2024) included AI knowledge as one of six independent dimensions in the TAICS scale, indicating that it has come to be recognized as a necessary component of teachers’ AI competence. Hava and Babayiğit (2024), in turn, identified a marked gap between teachers’ technical proficiency with AI tools and their actual instructional application of them, suggesting that tool-operation training alone is insufficient to develop competence and that teachers must also exercise judgment regarding AI’s capability boundaries.

### 2.5 Teacher self-efficacy theory

Self-efficacy refers to an individual’s belief about whether they can successfully accomplish a given task. Bandura (1997) argued that efficacy beliefs regulate behavior through cognitive, motivational, affective, and choice processes, and that they are formed primarily through four sources of information: enactive mastery experience, vicarious experience, verbal persuasion, and physiological and affective states. In AI-education contexts, AI self-efficacy has been shown to be a key predictor of teachers’ technology adoption: it mediates the relationship between AI attitudes and AI-TPACK (Mustafa et al., 2025; Dong et al., 2024; Durak, 2021), and increases in technology acceptance do not necessarily translate into corresponding growth in self-efficacy (Okumuş and Savas, 2024), suggesting that self-efficacy must be treated as an independent component. For the present study, self-efficacy theory explains the psychological mechanism through which teachers’ intrinsic beliefs about AI-empowered visualization teaching are externalized into developmental behaviors such as sustained reflection, professional planning, and collaborative exchange.

Accordingly, AI-VTC is not an extension of any single one of these literature streams, but rather a construct that integrates them within AI-empowered instructional contexts: it takes AI knowledge as its technological prerequisite, visualization teaching competency and visual instructional design as its substantive content, and teacher self-efficacy as the mechanism accounting for its sustained development, and it points to the integrated competence teachers require under conditions in which visual information is generated by AI. Unlike classical TPACK, which treats technology, content, and pedagogy as parallel knowledge components, this construct emphasizes the coordination of the three within authentic AI-mediated instructional tasks—teachers both draw on AI to generate visual representations and subject those representations to critical interpretation and pedagogical reconfiguration, reaching final instructional judgments through human–machine collaboration. This theoretical understanding provides the basis for the subsequent development of the scale’s dimensions and items.

## 3 Method

### 3.1 Scale development process

This study follows the logical chain of “theoretical construction-instrument development-reliability and validity validation.” First, integrating the AI-TPACK framework with teacher self-efficacy theory, it constructs a theoretical model within the three-parameter framework of “AI knowledge, visual instructional design and instructional self-efficacy”; second, in accordance with classical scale development methodology (Boateng et al., 2018), it formulates the initial scale through literature analysis, teacher interviews, Delphi expert consultation, and pilot testing; finally, through empirical testing with a teacher population, it employs methods such as item analysis, exploratory and confirmatory factor analysis, reliability testing, and measurement invariance testing to comprehensively examine the measurement quality and cross-group applicability of AI-VTCS.

### 3.2 Scale structure and dimensions

#### 3.2.1 Dimension one: design and creation of AI-assisted visualized instructional content

This dimension falls under AI technology integration competency and captures the “production-side” capacity of teachers to transform abstract subject-matter knowledge into effective visual representations using generative AI, grounded theoretically in the AI-TPACK framework. TPACK conceives teachers’ technology integration competency as the product of interactions among technological knowledge (TK), pedagogical knowledge (PK), and content knowledge (CK) (Khine et al., 2016). Generative AI has shifted teachers from being technology users to designers who co-create with AI, expanding their technological knowledge from operating predefined functions to prompt design, tool evaluation and selection, and output assessment and iterative refinement—a shift to which AI-TPACK is precisely the theoretical response (Hava and Babayiğit, 2024).

From a cognitive-theory standpoint, the dual-channel and limited-capacity assumptions of multimedia learning suggest that effective visual representations can spatialize abstract conceptual relationships and thereby reduce extraneous cognitive load (Mayer, 2009). Yet the pedagogical payoff of visual representations is by no means automatic; teachers must select appropriate representational forms in light of knowledge type, instructional goals, and learner characteristics—a capacity known as representational fluency. In AI-empowered contexts, this dimension manifests at three levels. First, AI tool usage competency: through effective prompt design, teachers convert texts, concepts, and even students’ thought processes into visualizable materials, while also gauging the suitability of different AI visualization tools for particular instructional tasks. The crux lies in grasping the generative logic and capability boundaries of AI tools and making informed choices among them. Second, content optimization and restructuring competency: teachers conduct pedagogical evaluation and adjustment of AI-generated content, identifying and remedying problems that are technically correct but pedagogically inappropriate (e.g., ill-chosen chart types, redundant visual elements), and continuously iterating and refining instructional presentations on the basis of AI visualization feedback. Third, creative integration and differentiated design competency: building on AI-generated outputs, teachers infuse pedagogical content knowledge (PCK) to weave discrete materials into a coherent visual instructional narrative, achieving the leap from AI generating individual charts to teachers designing visual instructional sequences, and they can also tailor differentiated visual learning resources to varied proficiency levels (Chiu et al., 2024).

Taken together, this study defines this dimension as teachers’ capacity, anchored in the AI-TPACK knowledge structure, to use generative AI tools for the generation, pedagogical optimization, and creative integration of visual instructional materials; to translate abstract subject-matter concepts, complex data relationships, and thought processes into appropriate visual representations; and to design differentiated visual resources that respond to diverse learners.

#### 3.2.2 Dimension two: awareness and interpretation of AI-generated visual information

This dimension, also situated under AI technology integration competency, addresses “the reception–critical evaluation side” of teachers’ interaction with AI visualization tools—namely, their capacity to comprehend, critically assess, and pedagogically translate the diverse visual information output by AI systems. It is jointly grounded in visual literacy and data literacy theory.

Visual literacy, first proposed by Debes (1969), emphasizes an individual’s ability to understand, interpret, and evaluate visual information. Generative AI and learning analytics, however, have altered the premises of traditional visual literacy: the algorithmic logic and data sources behind AI-generated artifacts such as learner profiles, analytics reports, and knowledge graphs are often opaque to teachers, requiring visual literacy to expand from comprehending given visual information toward scrutinizing the generative mechanisms and credibility of AI-produced information. Vázquez-Ingelmo et al. (2024) note that the effective interpretation of data visualizations depends not only on general visual literacy but also on domain-specific knowledge, and teachers must integrate visual interpretation with pedagogical content knowledge (PCK). From a data literacy standpoint, the Data Literacy for Teaching (DLFT) framework advanced by Gummer and Mandinach (2015) defines it as teachers’ ability to collect, analyze, and interpret various forms of data and translate information into actionable pedagogical knowledge and practice. The learner profiles generated by AI learning analytics systems are typically built on cluster analysis, predictive modeling, and association mining, which raise methodological concerns such as sampling bias, statistical significance, and overfitting. Teachers cannot remain at the surface reading of what the chart shows; rather, they need critical data thinking—asking how the chart was produced, whether the data are trustworthy, and whether the conclusions travel—so as to spot common misleading tactics such as truncated vertical axes and the conflation of correlation with causation, and to judge whether AI inferences apply to their own classroom contexts.

In AI-empowered settings, this dimension unfolds across three levels. First, technical interpretation competency: teachers accurately grasp the meaning of data indicators embedded in AI visual information, synthesize judgments about students’ learning status from multiple data sources, identify key learning problems from learner profiles, and connect visual information with PCK to deepen pedagogical understanding. This entails reading basic statistical indicators, understanding visual encoding conventions, and translating visual information back into instructional problems. Second, critical scrutiny competency: teachers recognize potential biases in data presentation, algorithmic bias, sampling bias, and data limitations in AI-generated visual artifacts, and remain alert to the misleading labeling of particular student groups. Drawing on an STS perspective, Vázquez-Ingelmo et al. (2024) reveal that data visualizations are not neutral representational tools but rather shape how teachers see students through particular frames—score-centered profiles, for instance, may lead teachers to overlook affective states. Third, pedagogical translation competency: teachers convert the instructional insights yielded by critically examined visual information into concrete teaching strategies and action plans, rather than passively accepting AI outputs.

In sum, this study defines this dimension as teachers’ integrated capacity, anchored in visual and data literacy, to conduct technical interpretation, critical scrutiny, and pedagogical translation of AI-generated visual information.

#### 3.2.3 Dimension three: decision-making and collaboration in AI-empowered visualized instruction

This dimension falls under visual instructional design competency and serves as the core mediating and transformative component of the entire competency model. Grounded in Cognitive Load Theory and the Cognitive Theory of Multimedia Learning, it centers on human-AI collaborative instructional decision-making in classroom implementation settings.

Cognitive Load Theory, proposed by Sweller (1988), distinguishes among intrinsic cognitive load (stemming from the inherent complexity of learning materials), extraneous cognitive load (arising from suboptimal instructional presentation), and germane cognitive load (generated through learners’ deep processing). Effective visual design reduces extraneous load by optimizing information presentation, thereby freeing cognitive resources for deep processing aligned with learning goals. The Cognitive Theory of Multimedia Learning, systematically developed by Mayer (2009), posits three core assumptions—dual-channel, limited capacity, and active processing—and distills twelve multimedia design principles, including the coherence principle, signaling principle, and spatial contiguity principle, providing a cognitive-science foundation for the design and evaluation of visual instructional materials. Both theories, however, predate the widespread classroom adoption of AI and rest on the assumption that the teacher is the sole designer and decision-maker of visual materials. With the advent of generative AI, systems can automatically generate instructional suggestions, recommend visualization schemes, and predict learning difficulties based on learner data, requiring teachers to redefine decision-making authority and responsibility within human-AI collaboration.

In AI-empowered classroom contexts, this dimension manifests at three levels. First, data-driven instructional decision-making competency: teachers independently make final instructional decisions based on AI-generated visual learning analytics, dynamically adjust classroom strategies in response to real-time data, and translate aggregated analytics (e.g., class-level knowledge mastery heatmaps, individual learning path tracking) into concrete instructional actions rather than passively relying on AI recommendations. Second, awareness of human-AI collaboration boundaries: teachers clearly delineate the division of authority and responsibility between humans and AI, discern discrepancies between AI suggestions and their own teaching experience and make reasoned choices, identify contextual factors that AI analytics systems cannot capture and factor them into decisions, weigh the risks and benefits of alternative instructional decisions when AI information is uncertain or incomplete, and preserve their professional agency. Vázquez-Ingelmo et al. (2024) underscore the critical role of domain knowledge in data visualization, suggesting that AI plays only the part of an intelligent assistant in instructional decision-making; when AI recommendations conflict with teachers’ professional judgment, teachers should have both the capacity and the responsibility to make the final call. Third, differentiated instructional intervention competency: teachers formulate differentiated intervention plans adapted to classroom contexts and students’ cognitive levels on the basis of AI visual data, and select appropriate visual representational forms and presentation pacing according to varying instructional content and learner characteristics. Hava and Babayiğit (2024) found a notable gap between teachers’ technical proficiency with AI tools and their actual pedagogical application, with instructional design competency serving as the key mediating variable that bridges this divide.

Taken together, this study defines this dimension as teachers’ core transformative capacity—anchored in cognitive load management principles and the cognitive principles of multimedia learning—to make data-driven instructional decisions within human-AI collaboration, clarify the division of human-AI authority and responsibility, and design differentiated instructional interventions.

#### 3.2.4 Dimension four: ethics and safety in AI-empowered visualized instruction

This dimension, situated under AI technology integration competency, is a regulatory competency that governs technology application throughout the entire workflow of using AI visualization tools. It is grounded in the ethical dimension of the AI-TPACK framework while extending the connotation of traditional AI ethics to the specific context of visualization teaching.

The TAICS scale developed by Chiu et al. (2024) lists AI ethics as one of six independent dimensions, indicating that ethical competency has become widely recognized as an integral part of teachers’ AI competency system. Nevertheless, existing AI ethics frameworks mostly articulate principled requirements at a macro level (e.g., fairness, transparency, accountability, privacy), lacking operational definitions of ethical risks specific to visualization teaching contexts. AI-empowered visualization teaching carries distinctive ethical sensitivity: on the one hand, AI visualization tools process large-scale student data (academic performance, learning behaviors, affective states, etc.), posing significantly higher risks of privacy breaches than conventional instruction; on the other hand, AI-generated visual artifacts (e.g., learner profiles, competency radar charts) exert strong framing and labeling effects, potentially entrenching stereotypes about particular groups through seemingly objective visual presentations. This means that ethical competency in visualization teaching cannot remain at the level of awareness of macro-level principles; it must be translated into the ability to identify, anticipate, and mitigate concrete ethical risks.

Within the specific context of AI-empowered visualization teaching, this dimension unfolds across three levels. First, privacy protection awareness: when using AI visualization tools to process student data, teachers can identify potentially private student information involved, understand the data flow mechanisms (what data are collected, where they are transmitted, and for what purposes), and adhere to ethical norms such as data minimization, purpose limitation, and informed consent. Second, algorithmic bias awareness: teachers can detect algorithmic bias or discriminatory representations potentially embedded in AI-generated visual artifacts and remain vigilant against their misleading labeling of particular student groups—bias may stem from under-representative training data, implicit assumptions in algorithm design (e.g., using test scores as the sole proxy for competency), or selective framing in visual presentation, and teachers should avoid relying on biased visual information as the sole basis for instructional decisions. Third, educational ethical judgment and guidance: teachers uphold the bottom line of educational ethics in AI visualization teaching practice; when visual artifacts contain discriminatory implications, data usage infringes upon students’ rights, or AI recommendations violate the principle of educational equity, teachers can weigh alternative choices within ethical dilemmas and make responsible decisions, while also guiding students to view AI-generated visual information critically. The UNESCO Global Framework of Professional Teaching Competence for AI in Education (Schmid et al., 2020) designates AI ethics as a core competency area, yet it leans toward the macro-policy level. This dimension responds precisely to this gap by translating abstract ethical requirements into the concrete practice of visualization teaching.

In summary, this study defines this dimension as teachers’ regulatory competency—encompassing privacy protection awareness, algorithmic bias awareness, and educational ethical judgment and guidance—to conduct data usage and visualization teaching in adherence to educational ethical norms throughout the entire workflow of AI visualization tool use.

#### 3.2.5 Dimension five: professional development competency in AI-empowered visualized instruction

This dimension falls under the theoretical dimension of AI teaching self-efficacy. It represents the externalization of teachers’ internal subjective efficacy beliefs into sustained professional development behaviors, with Bandura’s Self-Efficacy Theory as its core framework.

Self-efficacy refers to individuals’ beliefs about their capability to successfully accomplish a particular task. Bandura argued that efficacy beliefs regulate human behavior by influencing cognitive, motivational, affective, and selection processes, and that their formation draws primarily on four sources of information: mastery experiences, vicarious experiences, verbal persuasion, and physiological and affective states (Bandura, 1997). In the context of AI-empowered visualization teaching, teachers’ self-efficacy is not a static personality trait but rather develops dynamically through these four pathways in the course of ongoing instructional practice. Recent research has repeatedly confirmed the pivotal role of AI self-efficacy in teachers’ technology adoption: Mustafa et al. (2025) found that AI self-efficacy mediates the relationship between AI attitudes and AI-TPACK; Dong et al. (2024) showed that AI topic discussion positively predicts AI job crafting behaviors through the mediating mechanism of AI self-efficacy; and Okumuş and Savas (2024) found that improvements in technology acceptance do not necessarily coincide with parallel growth in self-efficacy, suggesting that self-efficacy must be measured and cultivated as an independent dimension rather than being subsumed by technology acceptance or technological competency.

Internal subjective efficacy beliefs externalize into teachers’ sustained professional development behaviors, and this dimension operationalizes precisely that externalization. It manifests at three levels. First, teaching reflection competency: teachers draw on AI visualization-related feedback to continuously reflect on and improve their own instructional practice, evaluating the effectiveness and shortcomings of AI-empowered visualization teaching. This corresponds to the mastery experiences source in Self-Efficacy Theory, as teachers develop an accurate understanding of their own capabilities by reflecting on both successful and unsuccessful experiences. Second, professional development planning competency: based on reflection outcomes and the instructional effectiveness assessments provided by AI learning analytics systems, teachers set their own professional development goals and proactively explore and experiment with new AI visualization teaching strategies to improve instructional outcomes, rather than passively awaiting external training. Research by Su (2024a, 2024b) demonstrates that structured training can significantly enhance AI self-efficacy, indicating that this dimension is amenable to intervention; however, the ultimate impact of training hinges on whether teachers translate it into proactive professional development planning and sustained practice. Third, collaborative communication competency: teachers share their experiences of AI-empowered visualization teaching with colleagues and foster collective growth through collaborative exchange within professional communities. This corresponds to the vicarious experiences and verbal persuasion sources in Self-Efficacy Theory—observing colleagues’ successful practices provides vicarious experiences, while feedback and encouragement within the community constitute verbal persuasion. It should be noted that this dimension measures the overt professional development behaviors driven by efficacy beliefs, rather than the subjective efficacy beliefs themselves. This design both preserves the theoretical foundation of Self-Efficacy Theory and ensures the observability and behavioral orientation of the scale items.

In summary, this study defines this dimension as teachers’ capacity for sustained professional development behavior—driven by AI teaching self-efficacy—to engage in teaching reflection through AI visualization-related feedback, plan professional development goals, and collaborate and communicate with colleagues.

### 3.3 Research design

This study adopted a cross-sectional survey design and followed the classical scale development procedure (Boateng et al., 2018). Two independent samples were used to conduct the exploratory factor analysis (EFA) and the confirmatory factor analysis (CFA), respectively, so as to avoid overfitting the factor structure to the same set of data. Data analysis was performed using SPSS 25.0 and Mplus 7.4: SPSS 25.0 was used for item analysis, normality testing, internal consistency and split-half reliability testing, criterion-related validity testing, and EFA; Mplus 7.4 was used for CFA and for testing composite reliability, convergent validity (AVE), discriminant validity, and cross-group measurement invariance. Model fit was evaluated comprehensively using indices such as χ^2^/df, RMSEA, CFI, TLI, and SRMR.

### 3.4 Sampling

This study adopted a convenience sampling method and selected primary and secondary school teachers in western China as the research participants. The sample was drawn from five provinces and regions—Shaanxi, Inner Mongolia, Hebei, Shanxi, and Anhui—covering teachers across all subject areas (e.g., Chinese, mathematics, English, science, and other subjects). Data were collected through the online platform “Wenjuanxing” (Questionnaire Star). To ensure the validity and reliability of the data, the questionnaire system set a minimum response time of 250 s to exclude careless responses. The research strictly followed academic ethical norms: before administration, all participants were fully informed of the study purpose, procedures, and the use of their data, and participated voluntarily under the principle of informed consent. Personal information was strictly anonymized, and it was assured that the data would be used solely for this academic analysis and never disclosed to third parties, thereby safeguarding participants’ privacy and data security.

### 3.5 Sample characteristics

A total of 591 questionnaires were collected. After rigorous screening and the removal of invalid responses (e.g., those with excessively short response times or patterned answering), 566 valid questionnaires were retained, yielding an effective response rate of 95.8%. The demographic characteristics of the sample were as follows: in terms of gender, there were 170 male teachers (30.0%) and 396 female teachers (70.0%); in terms of school type, 468 teachers (82.7%) were from public schools and 98 (17.3%) from private schools; in terms of educational stage, 201 teachers (35.5%) were from primary schools, 201 (35.5%) from middle schools, and 164 (29.0%) from high schools. Regarding years of teaching experience, teachers were categorized into five groups—1–5, 6–10, 11–15, 16–20, and 21 years or above—with the 6–10 and 11–15 year groups accounting for the largest proportions. The sample covered teachers from all subject areas, although the number of teachers differed across educational stages. Overall, the sample structure was reasonably balanced and possessed a certain degree of representativeness.

### 3.6 Instruments

#### 3.6.1 The AI-empowered visualization teaching competency scale (AI-VTCS)

Based on the definition of the core construct and relevant literature, this study took AI technology integration competency, visual instructional design competency, and AI teaching self-efficacy as its theoretical foundation, and operationalized AI-VTC into five observable dimensions—namely, the design and creation of AI-assisted visualized instructional content, awareness and interpretation of visual information, instructional decision-making and collaboration, ethics and safety, and professional development—thereby forming a three-level hierarchical structure of “theoretical dimensions–measurement dimensions–measurement items”.

Within this structure, Dimension 1 (Design and Creation), Dimension 2 (Awareness and Interpretation), and Dimension 4 (Ethics and Safety) belong to AI technology integration competency, characterizing teachers’ interaction with AI visualization tools from three perspectives—production, reception–evaluation, and constraint, respectively. Dimension 3 (Decision-Making and Collaboration) belongs to visual instructional design competency and constitutes the core link through which technological competence is transformed into instructional output. Dimension 5 (Professional Development) belongs to AI teaching self-efficacy and represents the persistent developmental behavior through which efficacy beliefs are externalized.

The five dimensions form a progressive and cyclical competency chain: Dimensions 1 and 2 serve as the foundation, Dimension 3 is the core of transformation, Dimension 4 provides ethical constraints throughout the entire process, and Dimension 5 drives the continuous improvement of the first four dimensions by running through the whole development process via teaching reflection, professional planning, and collaborative exchange. This design responds to the finding of Chiu et al. (2024) on the multidimensional nature of AI competency, while also avoiding the limitation of the TAICS six-dimension parallel structure, which lacks internal logical progression.

To align with actual teaching practice, the study adopted convenience sampling and conducted semi-structured interviews with 12 teachers in Xi’an (6 male and 6 female), based on which an initial item pool of 36 items was compiled. Five experts in educational technology and key teachers were then invited to evaluate the content validity and clarity of the items; five items with weak relevance or unclear wording were removed, yielding a 31-item pre-test questionnaire. The scale used a Likert 5-point scoring format (1 = strongly disagree, 5 = strongly agree), with higher scores indicating stronger AI-empowered visualization teaching competency.

## 4 Results

### 4.1 Common method bias test

Harman’s single-factor test was used to examine common method bias. All items of the self-developed scale and the criterion instruments were entered into an exploratory factor analysis using unrotated principal component analysis. The results showed that nine factors with eigenvalues greater than 1 were extracted, and the first factor accounted for 27.986% of the total variance, which is below the 40% threshold. This indicates that there is no serious common method bias in the present data, and that the influence of common source variance on the study’s results is generally limited.

### 4.2 Item analysis

Item analysis was conducted on the full sample, employing the critical ratio (CR) method and the item-total correlation method to assess item discrimination. After ranking the total scale scores in ascending order, the top and bottom 27% of participants were selected to form the high-scoring and low-scoring groups, respectively, and independent samples t-tests were carried out. The results (Table 1) showed that all items differed highly significantly between the two groups (t > 3.000, *p* < 0.001), with Cohen’s d values all exceeding 0.80 (large effect size), indicating satisfactory discriminatory power. In addition, item-total correlation analysis revealed that every item was significantly and positively correlated with the total scale score (across all 31 items), with correlation coefficients reaching the excellent threshold of r1 and r2 > 0.400, further corroborating the items’ discriminative ability. In sum, all items met the retention criteria and were kept; the subsequent exploratory factor analysis (EFA), confirmatory factor analysis (CFA), and reliability and validity examinations in this study were all conducted on the full set of 31 items.

**Table 1:** Item analysis results of the scale (*n* = 566).

Item	High-score group (M(SD))	Low-score group (M(SD))	*t*	*d*	*r_1_*	*r_2_*
A1	3.75 (0.927)	2.71 (0.887)	10.301^***^	1.147	0.475^***^	0.473^***^
A2	3.96 (0.785)	2.81 (0.843)	12.683^***^	1.411	0.598^***^	0.571^***^
A3	3.93 (0.695)	2.46 (0.663)	19.485^***^	2.174	0.677^***^	0.678^***^
A4	4.06 (0.599)	2.70 (0.745)	18.152^***^	2.011	0.696^***^	0.669^***^
A5	3.96 (0.648)	2.52 (0.681)	19.431^***^	2.163	0.668^***^	0.669^***^
A6	4.01 (0.573)	2.58 (0.702)	20.133^***^	2.232	0.736^***^	0.707^***^
A7	4.03 (0.543)	2.58 (0.751)	20.023^***^	2.213	0.735^***^	0.724^***^
A8	4.00 (0.562)	2.49 (0.646)	22.404^***^	2.488	0.768^***^	0.752^***^
A9	3.91 (0.566)	2.65 (0.716)	17.570^***^	1.946	0.705^***^	0.690^***^
A10	4.00 (0.500)	2.85 (0.713)	16.947^***^	1.872	0.693^***^	0.669^***^
A11	4.02 (0.590)	2.66 (0.654)	19.662^***^	2.185	0.729^***^	0.724^***^
A12	4.02 (0.466)	2.63 (0.623)	22.850^***^	2.527	0.781^***^	0.768^***^
A13	4.03 (0.531)	2.68 (0.683)	19.939^***^	2.207	0.746^***^	0.742^***^
A14	4.02 (0.532)	2.62 (0.616)	21.951^***^	2.437	0.758^***^	0.753^***^
A15	4.10 (0.447)	2.70 (0.594)	24.152^***^	2.672	0.791^***^	0.787^***^
A16	4.07 (0.509)	2.74 (0.667)	20.270^***^	2.243	0.743^***^	0.728^***^
A17	4.07 (0.482)	2.78 (0.666)	19.968^***^	2.207	0.757^***^	0.718^***^
A18	4.05 (0.470)	2.82 (0.659)	19.529^***^	2.158	0.766^***^	0.730^***^
A19	4.06 (0.462)	2.68 (0.757)	19.936^***^	2.195	0.731^***^	0.713^***^
A20	4.05 (0.410)	2.66 (0.672)	22.747^***^	2.504	0.763^***^	0.749^***^
A21	3.99 (0.506)	2.67 (0.660)	20.323^***^	2.249	0.746^***^	0.734^***^
A22	4.07 (0.670)	2.79 (0.768)	15.964^***^	1.773	0.680^***^	0.667^***^
A23	4.09 (0.478)	2.85 (0.719)	18.397^***^	2.029	0.747^***^	0.712^***^
A24	4.01 (0.579)	2.75 (0.785)	16.514^***^	1.826	0.691^***^	0.673^***^
A25	4.04 (0.572)	2.81 (0.764)	16.523^***^	1.828	0.708^***^	0.675^***^
A26	4.15 (0.497)	2.97 (0.780)	16.369^***^	1.804	0.703^***^	0.666^***^
A27	4.12 (0.458)	2.96 (0.749)	16.962^***^	1.867	0.685^***^	0.673^***^
A28	4.09 (0.435)	2.87 (0.718)	18.688^***^	2.057	0.726^***^	0.702^***^
A29	4.08 (0.544)	2.84 (0.700)	17.828^***^	1.974	0.736^***^	0.690^***^
A30	4.18 (0.488)	3.00 (0.792)	16.241^***^	1.788	0.699^***^	0.656^***^
A31	4.18 (0.488)	2.94 (0.767)	17.438^***^	1.921	0.703^***^	0.677^***^

***p < 0.001, **p < 0.01,*p < 0.05. r_1_ = Pearson correlation coefficient; r_2_ = Spearman correlation coefficient.

### 4.3 Split-half grouping and normality testing

For cross-validation purposes, the full sample was randomly divided into two equal halves, designated for exploratory factor analysis (EFA) and confirmatory factor analysis (CFA), respectively. Meanwhile, to determine the appropriate analytical methods for subsequent procedures, normality tests were performed on each of the two subsamples using SPSS 25.0, based on indicators including skewness and kurtosis (see Table 2).

**Table 2:** Normality test of the survey data for the scale (*n* = 566).

Item	First half of the sample (*n* = 283)					Second half of the sample (*n* = 283)				
	M	SD	Sk	Ku	Nor	M	SD	Sk	Ku	Nor
A1	3.24	0.914	−0.495	0.117	Y	3.19	0.952	−0.266	−0.041	Y
A2	3.31	0.848	−0.572	0.610	Y	3.37	0.911	−0.368	−0.061	Y
A3	3.18	0.857	−0.178	−0.166	Y	3.11	0.931	−0.068	−0.388	Y
A4	3.30	0.789	−0.195	0.148	Y	3.35	0.864	−0.277	0.171	Y
A5	3.26	0.839	−0.126	−0.222	Y	3.13	0.903	−0.145	−0.232	Y
A6	3.30	0.789	−0.195	0.148	Y	3.20	0.878	−0.088	−0.128	Y
A7	3.32	0.824	−0.305	0.032	Y	3.21	0.890	−0.186	−0.176	Y
A8	3.25	0.757	−0.264	0.193	Y	3.19	0.908	−0.136	−0.299	Y
A9	3.29	0.776	−0.363	0.024	Y	3.23	0.799	−0.312	0.149	Y
A10	3.39	0.737	−0.440	0.296	Y	3.43	0.775	−0.493	0.625	Y
A11	3.30	0.815	−0.327	0.220	Y	3.31	0.806	−0.222	0.034	Y
A12	3.28	0.742	−0.403	−0.051	Y	3.31	0.801	−0.212	−0.137	Y
A13	3.36	0.793	−0.532	0.454	Y	3.34	0.811	−0.414	0.302	Y
A14	3.36	0.771	−0.262	0.127	Y	3.28	0.801	−0.255	−0.062	Y
A15	3.42	0.756	−0.324	0.316	Y	3.39	0.798	−0.361	0.179	Y
A16	3.43	0.761	−0.421	0.266	Y	3.42	0.797	−0.479	−0.007	Y
A17	3.48	0.721	−0.630	0.653	Y	3.45	0.786	−0.371	−0.051	Y
A18	3.50	0.721	−0.679	1.023	Y	3.45	0.758	−0.256	0.110	Y
A19	3.39	0.810	−0.702	0.697	Y	3.36	0.824	−0.420	0.074	Y
A20	3.36	0.743	−0.388	0.221	Y	3.36	0.820	−0.373	−0.055	Y
A21	3.33	0.749	−0.459	0.272	Y	3.31	0.804	−0.201	0.048	Y
A22	3.46	0.790	−0.483	0.353	Y	3.41	0.896	−0.407	−0.127	Y
A23	3.50	0.711	−0.589	0.774	Y	3.48	0.814	−0.574	0.445	Y
A24	3.37	0.803	−0.400	0.266	Y	3.34	0.877	−0.488	0.015	Y
A25	3.43	0.784	−0.314	0.164	Y	3.44	0.837	−0.533	0.326	Y
A26	3.55	0.715	−0.446	0.548	Y	3.56	0.816	−0.576	0.845	Y
A27	3.59	0.705	−0.519	1.088	Y	3.49	0.792	−0.542	0.425	Y
A28	3.53	0.726	−0.737	0.785	Y	3.57	0.757	−0.830	1.024	Y
A29	3.49	0.736	−0.654	0.883	Y	3.52	0.791	−0.616	0.755	Y
A30	3.63	0.730	−0.663	0.764	Y	3.63	0.795	−0.686	1.053	Y
A31	3.59	0.730	−0.674	0.962	Y	3.62	0.792	−0.719	0.849	Y

### 4.4 Exploratory factor analysis

Exploratory factor analysis (EFA) was performed on one half of the sample (*n* = 283) using SPSS 25.0. The results yielded a KMO value of 0.945 and a significant Bartlett’s test of sphericity (χ^2^ = 7248.268, *p* < 0.001), indicating sufficient inter-item correlations and that the data were highly suitable for factor analysis. Principal axis factoring was employed for common factor extraction, followed by orthogonal rotation with varimax. Items were removed iteratively based on the following criteria: factor loading < 0.45; measure of sampling adequacy (MSA) < 0.80; significant cross-loadings; or misalignment between item assignment and the theoretical construct. Furthermore, retained factors were required to have eigenvalues > 1 and to comprise at least three items each. As shown in Table 3, the factor structure extracted by EFA was largely consistent with the dimensional structure specified during scale development (Brandenburg, 2024). Despite the stringent statistical screening thresholds applied, all 31 items satisfied the criteria with respect to factor loadings, MSA values, and theoretical consistency. Consequently, no items were removed and the original factor labels were retained; the subsequent confirmatory factor analysis (CFA) and structural equation modeling (SEM) analyses were all conducted on the full 31-item scale.

**Table 3:** Exploratory factor analysis results of the scale (*n* = 283).

Item	Factor loading	MSA	Rotated factor loadings				
			1	2	3	4	5
A1	0.545	0.945^a^	**0.689**				
A2	0.702	0.929^a^	**0.789**				
A3	0.683	0.949^a^	**0.715**				
A4	0.696	0.960^a^	**0.717**				
A5	0.648	0.959^a^	**0.660**				
A6	0.622	0.963^a^	**0.611**				
A7	0.622	0.940^a^	**0.611**				
A8	0.634	0.959a	**0.592**				
A9	0.688	0.972^a^				**0.651**	
A10	0.710	0.956^a^				**0.682**	
A11	0.671	0.963^a^				**0.627**	
A12	0.709	0.955^a^				**0.590**	
A13	0.701	0.968^a^				**0.641**	
A14	0.694	0.955^a^				**0.627**	
A15	0.742	0.961^a^				**0.604**	
A16	0.692	0.957^a^			**0.695**		
A17	0.743	0.956^a^			**0.684**		
A18	0.735	0.960^a^			**0.662**		
A19	0.750	0.963^a^			**0.715**		
A20	0.751	0.955^a^			**0.729**		
A21	0.718	0.955^a^			**0.672**		
A22	0.706	0.957^a^		**0.747**			
A23	0.791	0.955^a^		**0.748**			
A24	0.807	0.918^a^		**0.835**			
A25	0.782	0.943^a^		**0.780**			
A26	0.701	0.921^a^		**0.656**			
A27	0.726	0.932^a^		**0.667**			
A28	0.807	0.967^a^					**0.712**
A29	0.733	0.960^a^					**0.668**
A30	0.749	0.948^a^					**0.745**
A31	0.777	0.954^a^					**0.732**
Eigenvalue			16.107	2.303	1.333	1.232	1.058
Variance explained (%)			51.957	7.430	4.298	3.975	3.414
Cumulative variance explained (%)			51.957	59.387	63.685	67.660	71.074

Bold values indicate the primary factor loadings of each item on its corresponding factor. MSA = Measure of Sampling Adequacy. ^a^*p* < 0.001.

### 4.5 Confirmatory factor analysis

Confirmatory factor analysis (CFA) was performed on the other half of the sample (*n* = 283). First- and second-order measurement models were estimated using Mplus 7.4 (Figures 1, 2), with the weighted least squares mean and variance-adjusted (WLSMV) estimator. The criteria for evaluating model goodness of fit were established as follows: a χ^2^/df ratio below 3 was considered excellent fit and below 5 acceptable; both CFI and TLI were required to exceed 0.90; RMSEA and SRMR values below 0.05 were regarded as excellent fit and below 0.08 acceptable. If the fit indices fell short of these standards, the model was revised on the basis of modification indices (allowing covariances among measurement error terms) or by removing inappropriate items, until model fit reached an acceptable level.

**Figure 1:**
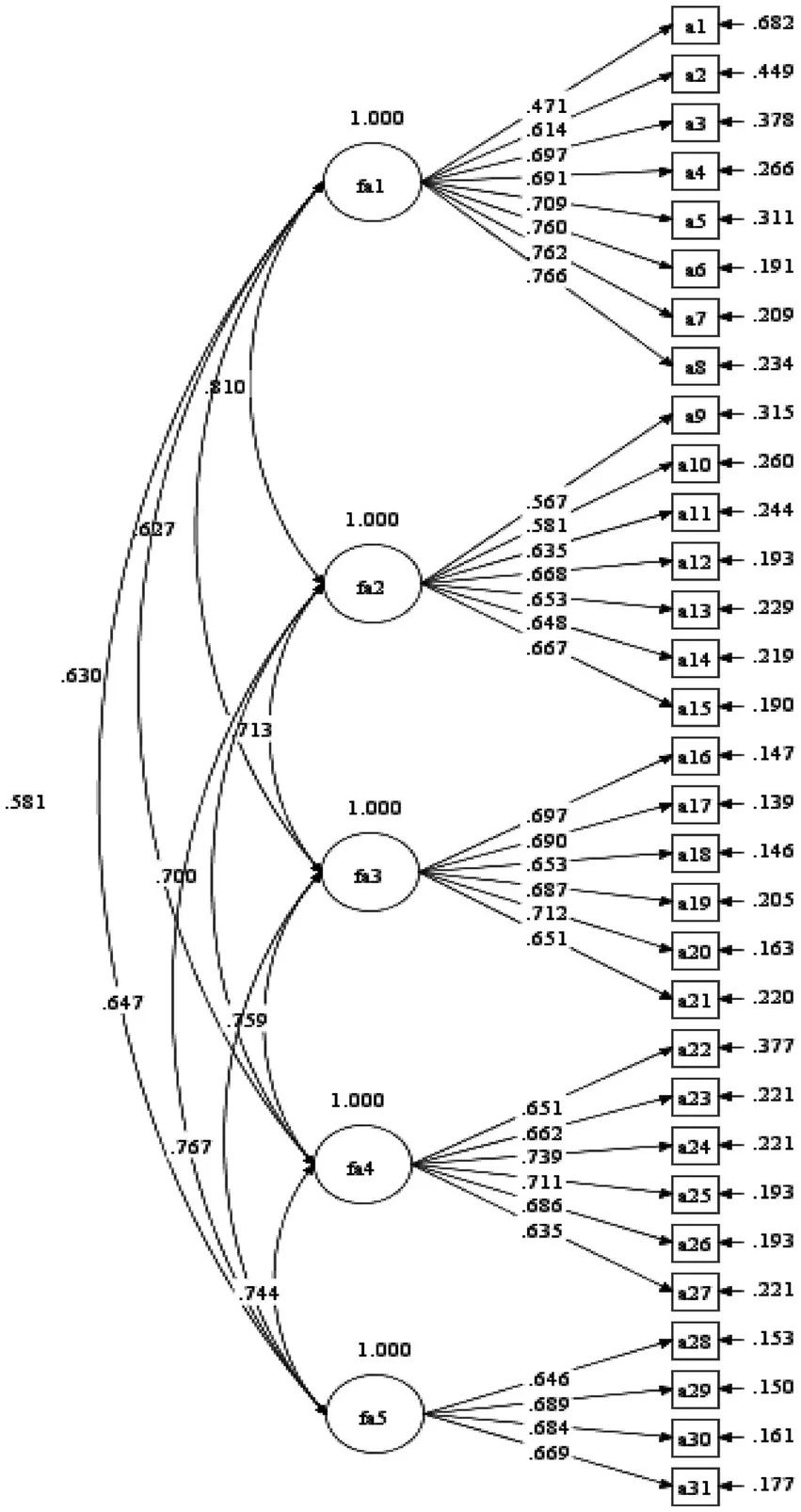
Confirmatory factor analysis results of the first order measurement model of the scale (*n* = 283).

**Figure 2:**
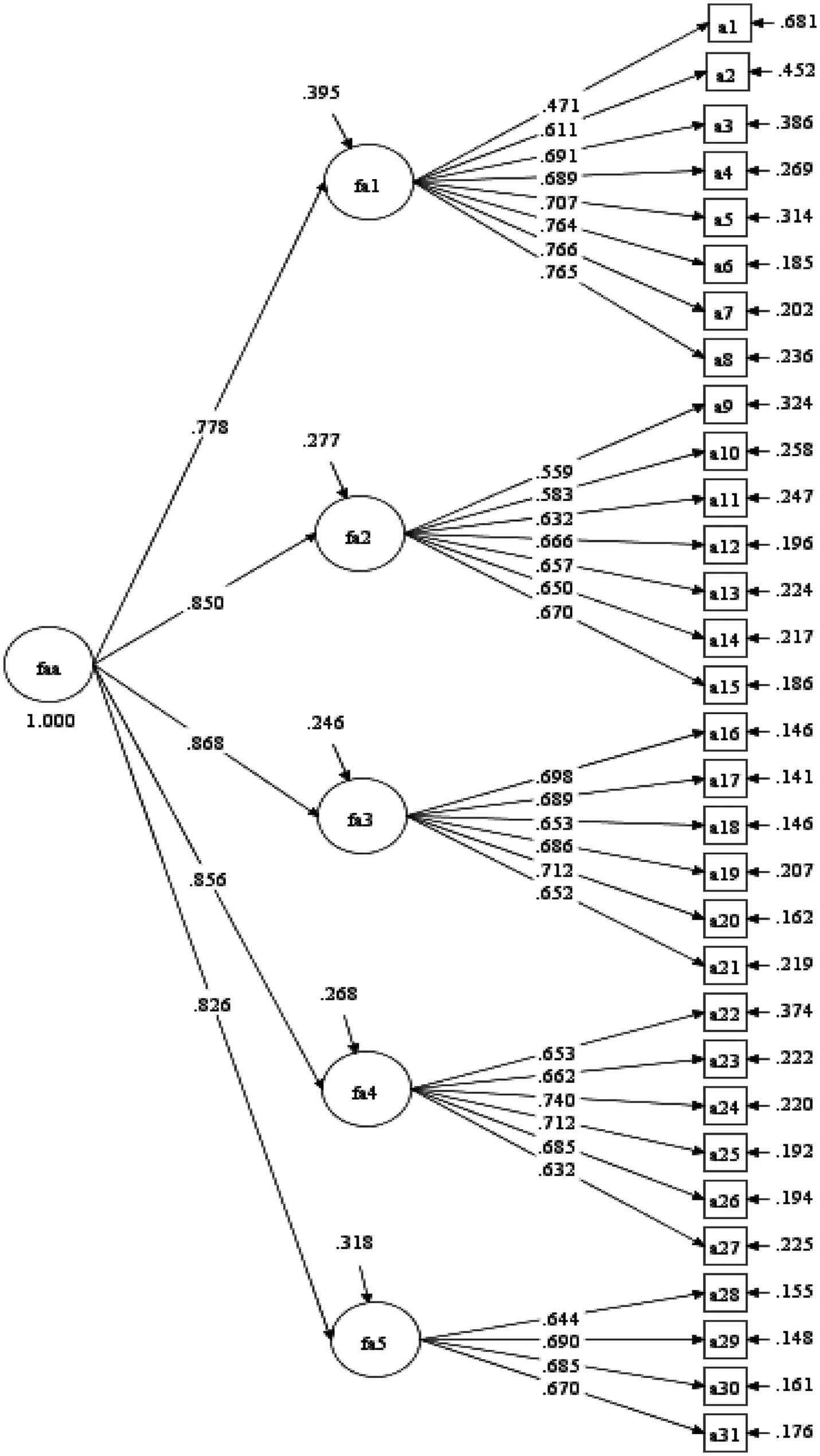
Confirmatory factor analysis results of the second order measurement model of the scale (*n* = 283).

Table 4 shows that both the first-order five-factor model (Figure 1) and the second-order five-factor model (Figure 2) met the recommended criteria for good fit: CFI and TLI values were both above 0.900, χ^2^/df values were below 3.00, and RMSEA and SRMR values were below 0.080. These results indicate that the scale possesses good structural validity, and all items were retained.

**Table 4:** Model fit indices for confirmatory factor analysis of the scale (*n* = 283).

Model	χ^2^	df	χ^2^/df	CFI	TLI	RMSEA (90% CI)	SRMR
First order	1045.281	424	2.465	0.918	0.910	0.072(0.066,0.077)	0.045
Second order	1107.843	427	2.594	0.910	0.902	0.075(0.069,0.080)	0.060

### 4.6 Composite reliability, convergent validity, and discriminant validity analyses

To further examine the reliability and validity of the scale, based on the full sample (*N* = 566), composite reliability, convergent validity, and discriminant validity were evaluated using Mplus 7.4 with the weighted least squares mean and variance adjusted (WLSMV) estimator. The evaluation criteria were as follows: (1) the squared standardized factor loading (i.e., the squared multiple correlation, SMC) was used as the item reliability index, and items with an SMC below 0.40 were considered for deletion; (2) a composite reliability (CR) above 0.70 and an average variance extracted (AVE) above 0.50 were taken as evidence of good convergent validity; and (3) according to the Fornell-Larcker criterion, discriminant validity was judged to be satisfactory when the square root of the AVE for each factor exceeded the absolute value of the correlation between that factor and the other factors.

The results of the standardized factor loadings, composite reliability (CR), average variance extracted (AVE), and discriminant validity (DV) tests for the first-order five-factor model (Figure 1) and the second-order five-factor model (Figure 2) are presented in Table 5.

**Table 5:** Composite reliability, convergent validity, and discriminant validity of the scale (*n* = 566).

Model	DIM	IR	CR	CV	DV				
		Std. loading	CR	AVE	F1	F2	F3	F4	F5
First order	F1	0.528–0.833	0.915	0.576	**0.759**				
	F2	0.749–0.837	0.925	0.638	0.812	**0.799**			
	F3	0.817–0.855	0.935	0.704	0.664	0.768	**0.839**		
	F4	0.757–0.843	0.923	0.666	0.605	0.714	0.759	**0.816**	
	F5	0.829–0.864	0.951	0.829	0.625	0.707	0.768	0.730	**0.911**
Second order	F1	0.528–0.836	0.914	0.571	**0.755**				
	F2	0.745–0.838	0.925	0.638	0.711	**0.799**			
	F3	0.818–0.854	0.935	0.705	0.704	0.787	**0.839**		
	F4	0.759–0.842	0.923	0.666	0.660	0.737	0.730	**0.816**	
	F5	0.829–0.864	0.911	0.720	0.665	0.743	0.735	0.689	**0.849**

IR = Item reliability; CR = Composite reliability; CV=Convergent validity; DV: discriminant validity; Bold diagonal values are the square roots of the AVE; values below the diagonal are Pearson correlation coefficients between factors. The same applies to subsequent tables.

Regarding convergent validity, in the first-order model, the standardized loadings of all items ranged from 0.528 to 0.864, all exceeding 0.50 and reaching statistical significance. The CR values of the five dimensions ranged from 0.915 to 0.951, all exceeding 0.70, and the AVE values ranged from 0.576 to 0.829, all exceeding 0.50. The results of the second-order model were similar: the standardized loadings of all items ranged from 0.528 to 0.864, the CR values of the dimensions ranged from 0.911 to 0.935, and the AVE values ranged from 0.571 to 0.720, all meeting the corresponding criteria. These results indicate that all dimensions in both models demonstrated good convergent validity.

Regarding discriminant validity, in the second-order model, the square roots of the AVE values for each dimension (0.755–0.849, shown in bold on the diagonal) were all greater than the correlations between that dimension and the other dimensions (0.660–0.787), satisfying the Fornell-Larcker criterion. In the first-order model, all pairs of dimensions satisfied this criterion except for F1–F2 (r = 0.812); the high correlation between these two dimensions suggests that their variance may be accounted for by a higher-order common factor. After introducing the second-order factor, all dimensions satisfied the Fornell-Larcker criterion, indicating that the second-order structure is more appropriate for the data and that the scale as a whole possesses good discriminant validity.

### 4.7 Measurement invariance testing

To further examine the applicability of the scale, measurement invariance was tested through multigroup confirmatory factor analysis on the full sample using Mplus7.4 with the weighted least squares mean and variance-adjusted (WLSMV) estimation. Specifically, four models were constructed in sequence—configural invariance (Model A), metric invariance (Model B), scalar invariance (Model C), and strict invariance (Model D)—to investigate the measurement invariance of the AI-Empowered Teacher Visualization Teaching Competency Scale across gender groups, school types (public and private), and teaching stages (primary, middle, and high school). In nested model comparisons, measurement invariance was considered established if ΔCFI < 0.010 and ΔRMSEA < 0.015, with ΔTLI < 0.010 and ΔSRMR < 0.030 serving as supplementary criteria. As shown in Table 6, the scale demonstrated satisfactory measurement invariance both between male and female teachers and between public and private school and different teaching stages.

**Table 6:** Measurement invariance tests of the scale across gender, school type, and teaching stage (*n* = 566).

Model	χ^2^	df	TLI	CFI	SMRM	RMSEA (90% CI)	MC	ΔCFI	ΔRMSEA
Invariance across gender (male, female)									
A	2091.903	848	0.916	0.908	0.041	0.072(0.068,0.076)			
B	2118.246	874	0.916	0.910	0.046	0.071(0.067,0.075)	B vs. A	0.002	−0.001
C	2153.149	900	0.915	0.912	0.046	0.070(0.066,0.074)	C vs. B	0.002	−0.001
D	2318.982	931	0.906	0.906	0.054	0.073(0.069,0.076)	D vs. C	−0.006	0.003
Invariance across school (public, private)									
A	2023.855	848	0.920	0.912	0.042	0.070(0.066,0.074)			
B	2065.584	874	0.919	0.914	0.051	0.069(0.066,0.073)	B vs. A	0.002	−0.001
C	2098.059	900	0.918	0.916	0.051	0.069(0.065,0.072)	C vs. B	0.002	0.000
D	2208.191	931	0.913	0.913	0.055	0.070(0.066,0.073)	D vs. C	−0.003	0.001
Invariance across teaching stage (primary, middle, and high school)									
A	2621.257	1,257	0.910	0.900	0.047	0.076(0.072,0.080)			
B	2664.794	1,309	0.911	0.905	0.055	0.074(0.070,0.078)	B vs. A	0.005	−0.002
C	2725.564	1,361	0.910	0.908	0.056	0.073(0.069,0.077)	C vs. B	0.003	−0.001
D	2903.312	1,423	0.902	0.904	0.065	0.074(0.070,0.078)	D vs. C	−0.004	0.001

MC = Model comparison; Model A = configural invariance; Model B = metric invariance; Model C = scalar invariance; Model D = strict invariance.

### 4.8 Reliability testing

Based on the full sample, internal consistency reliability and split-half reliability tests were conducted on the scale using SPSS 25.0. In accordance with conventional psychometric standards, Cronbach’s α coefficients were interpreted as follows: > 0.900 indicated excellent, 0.800–0.899 good, and 0.700–0.799 acceptable. Split-half reliability was assessed using the Spearman-Brown coefficient, with the same threshold (≥ 0.700) applied to evaluate the equivalence and internal stability of the two halves of the scale. As shown in Table 7, the Cronbach’s α coefficients of the subscales ranged from 0.911 to 0.934, all below the overall scale coefficient of 0.967. Split-half reliability analysis yielded Cronbach’s α coefficients of 0.946 and 0.953 for the first and second halves (items 1–15 and items 16–31), respectively, with a Spearman-Brown coefficient of 0.860. These findings indicate that the AI-Empowered Teacher Visualization Teaching Competency Scale possesses satisfactory internal consistency reliability and split-half reliability.

**Table 7:** Reliability analysis results of the scale (*n* = 566).

Consistency reliability				Split-half reliability		
Factor	Item	Cronbach’s α	Cronbach’s α	Cronbach’s α-1	Cronbach’s α-2	Spearman-Brown coefficient
F1	8	0.913	0.967	0.946	0.953	0.860
F2	7	0.925				
F3	6	0.934				
F4	6	0.922				
F5	4	0.911				

### 4.9 Criterion-related validity testing

Based on the full sample, criterion-related validity testing was conducted using SPSS 25.0. As shown in Table 8, all five dimensions of the AI-Empowered Teacher Visualization Teaching Competency Scale were significantly and positively correlated with all seven dimensions of the Teacher Technological Pedagogical Content Knowledge (TPACK) Scale, with correlation coefficients ranging from 0.560 to 0.737 (all *p* < 0.001). This finding confirms that the scale possesses satisfactory criterion-related validity.

**Table 8:** Criterion-related validity analysis results (*n* = 566).

Dimension	PK	CK	TK	PCK	TPK	TCP	TPCK
DCC	0.631^***^	0.678^***^	0.671^***^	0.696^***^	0.692^***^	0.607^***^	0.644^***^
AIV	0.635^***^	0.699^***^	0.680^***^	0.737^***^	0.693^***^	0.666^***^	0.710^***^
DMC	0.613^***^	0.672^***^	0.650^***^	0.687^***^	0.647^***^	0.630^***^	0.677^***^
ES	0.608^***^	0.630^***^	0.608^***^	0.673^***^	0.655^***^	0.618^***^	0.644^***^
PDC	0.560^***^	0.622^***^	0.604^***^	0.679^***^	0.622^***^	0.585^***^	0.648^***^

## 5 Discussion and conclusion

This study aimed to develop and validate the AI-Empowered Teacher Visualization Teaching Competency Scale (AI-VTCS). Drawing on the AI-TPACK framework, visual literacy and data literacy theories, Cognitive Load Theory, the Cognitive Theory of Multimedia Learning, and Self-Efficacy Theory, the researchers constructed a five-dimensional theoretical framework and systematically evaluated the psychometric properties of the scale through item analysis, exploratory factor analysis (EFA), confirmatory factor analysis (CFA), reliability testing, measurement invariance testing, and criterion-related validity testing. The results indicate that AI-VTCS possesses good reliability and validity and can serve as an effective instrument for measuring teacher visualization teaching competency under AI empowerment.

In terms of psychometric properties, the item analysis revealed that all 31 items demonstrated satisfactory discrimination and difficulty (CR > 3.000, *p* < 0.001; Cohen’s d > 0.80; item-total correlations > 0.400). Item-total correlations were computed using both Pearson and Spearman coefficients, given the ordinal nature of the Likert-scale data. All items met the retention criteria and were therefore retained. Building on this, EFA extracted five factors with eigenvalues greater than 1 (KMO = 0.945; Bartlett χ^2^ = 7248.268, *p* < 0.001), accounting for a cumulative variance of 71.074%. All item loadings were no lower than 0.45 with no significant cross-loadings, and the factor structure aligned closely with the theoretical specification. Regarding reliability, the Cronbach’s α coefficients of the subscales ranged from 0.911 to 0.934, all below the overall scale coefficient of 0.967. and the Spearman-Brown split-half coefficient was 0.860, indicating excellent internal consistency. CFA further supported this five-factor structure: the first-order model fit well (χ^2^/df = 2.465, CFI = 0.918, TLI = 0.910, RMSEA = 0.072, SRMR = 0.045), with all standardized item loadings exceeding 0.50; the second-order model also achieved acceptable-to-good fit (χ^2^/df = 2.594, CFI = 0.910, TLI = 0.902, RMSEA = 0.075, SRMR = 0.060). Together with the moderate-to-high correlations among the five latent variables (r = 0.605–0.812), these findings support the existence of a higher-order competency construct. For cross-group comparisons, measurement invariance from configural to strict invariance was established across gender, school type (public/private), and teaching stage (primary, middle, and high school) (ΔCFI < 0.010, ΔRMSEA < 0.015), demonstrating cross-group comparability. Finally, validity evidence was equally robust: the five dimensions exhibited composite reliability of 0.915–0.951 and average variance extracted (AVE) of 0.576–0.829. In the second-order model, the square root of each factor’s AVE exceeded its correlations with all other factors, indicating good convergent and discriminant validity; the single pair not satisfying this criterion in the first-order model (F1–F2, r = 0.812) was accounted for by the higher-order structure. All dimensions of AI-VTCS were significantly and positively correlated with the seven dimensions of the TPACK scale (r = 0.560–0.737, *p* < 0.001), supporting criterion-related validity. Taken together, AI-VTCS met psychometric standards across item quality, factor structure, reliability, model fit, measurement invariance, and validity. These empirical findings not only confirm the reliability and validity of the scale but also provide data support for further elucidating the theoretical underpinnings of the five-dimensional framework.

From a theoretical standpoint, the five-dimensional framework proposed in this study essentially delineates the specific competency structure that teachers need to seek, comprehend, process, use, and evaluate visual instructional information in AI-empowered contexts. The Design and Creation dimension captures teachers’ production-side competency in using generative AI to transform abstract knowledge into effective visual representations, serving as the starting point of AI-empowered visualization teaching (Khine et al., 2016; Hava and Babayiğit, 2024). The Awareness and Interpretation dimension addresses the competency to technically interpret, critically scrutinize, and pedagogically translate AI-generated visual information, requiring teachers to move beyond surface-level reading and cultivate critical data thinking (Gummer and Mandinach, 2015; Vázquez-Ingelmo et al., 2024). The Decision-Making and Collaboration dimension functions as the core mediating and transformative component, emphasizing teachers’ preservation of agency within human-AI collaboration and their capacity to make independent instructional decisions based on AI learning analytics (Sweller, 1988; Mayer, 2009). The Ethics and Safety dimension is a regulatory competency encompassing privacy protection, algorithmic bias awareness, and educational ethical judgment, addressing the distinctive ethical risks in AI-empowered visualization teaching (Chiu et al., 2024; Schmid et al., 2024). The Professional Development dimension represents the sustained developmental behavior through which AI teaching self-efficacy is externalized—including teaching reflection, professional planning, and collaborative communication—and serves as the intrinsic driving force for continuous competency improvement (Bandura, 1997; Mustafa et al., 2025). These five dimensions are not isolated from one another; rather, they exhibit systematic patterns of interrelation in the empirical data.

Beyond confirming the significance of the associations, the differential strength of the criterion-related correlations warrants interpretation within the nomological network of the construct. Awareness and Interpretation and Decision-Making and Collaboration showed the strongest correlations with the TPACK dimensions (0.613–0.737), as both share the cognitive–evaluative core of TPACK—namely, the understanding and judgment of how technology, content, and pedagogy interact. By contrast, Professional Development exhibited the weakest correlations (0.560–0.648), consistent with its theoretical anchoring in self-efficacy-driven developmental behavior rather than in the knowledge-integration structure of TPACK. The moderate correlations of Design and Creation and Ethics and Safety (0.607–0.696) reflect components that extend beyond classical TPACK—generative output and ethical considerations—which are distinctive features of AI-empowered competency. Overall, this gradient indicates that AI-VTC is related to, yet distinct from, TPACK: it shares the technology-integration core while adding unique generative, ethical, and efficacy-driven elements, thereby clarifying its nomological network.

The conceptualization of a higher-order competency construct warrants further elaboration. Although the first-order model yielded slightly more favorable fit indices, the second-order model also met all recommended criteria for good fit, and the moderate-to-high correlations among the five latent variables (r = 0.605–0.812) indicate that the dimensions are substantially interrelated rather than independent. This pattern supports the notion that their variance may be accounted for by a common higher-order factor, namely overall AI-empowered visualization teaching competency—defined as teachers’ integrated capacity to seek, comprehend, process, use, and evaluate visual instructional information through AI across the full cycle of teaching. Within this broader construct, each first-order dimension operates as an interrelated component serving a distinct functional role: Design and Creation and Awareness and Interpretation constitute the foundational input and evaluation competencies; Decision-Making and Collaboration functions as the pivotal link translating technological capacity into instructional practice; Ethics and Safety runs through the entire process to provide cross-cutting regulatory constraints; and Professional Development supplies the efficacy-driven impetus for the continuous improvement of all dimensions. The hierarchical structure is thus supported not by a marginal difference in fit but by the substantive interrelatedness of the dimensions and their coherent organization under a unifying higher-order competency. On the basis of the above empirical and theoretical analyses, the overall conclusion of this study can be drawn.

Grounded in the AI-TPACK framework, visual literacy and data literacy theories, Cognitive Load Theory, the Cognitive Theory of Multimedia Learning, and Self-Efficacy Theory, this study conceptualizes AI-empowered teacher visualization teaching competency as a construct comprising five dimensions—Design and Creation, Awareness and Interpretation, Decision-Making and Collaboration, Ethics and Safety, and Professional Development—and accordingly developed the 31-item AI-VTCS. After systematic validation through item analysis, EFA, CFA, reliability testing, measurement invariance testing, convergent and discriminant validity testing, and criterion-related validity testing, the results demonstrate that AI-VTCS possesses good reliability and validity and can serve as an effective instrument for measuring teacher visualization teaching competency under AI empowerment. This study not only enriches theoretical research on teacher competency structures in the AI era but also provides a valuable measurement tool for assessing and enhancing teachers’ AI visualization teaching competency in teacher education practice.

## 6 Research implications

### 6.1 Theoretical implications

The theoretical contributions of this study are mainly reflected in two aspects. First, this study explicitly defines the concept of AI-empowered teacher visualization teaching competency and operationalizes it into a five-dimensional structure, thereby addressing the systematic lag in existing teacher competency frameworks. The traditional TPACK framework is rooted in the Web 2.0 era, with its technological connotation centered on interactive whiteboards and multimedia courseware, and it failed to anticipate the fundamental reshaping of instructional practice by generative AI (Eutsler, 2021); although the more recently developed TAICS scale covers dimensions such as AI knowledge, AI pedagogy, and AI ethics (Chiu et al., 2024), it likewise does not define visualization teaching competency as an independent construct. In this respect, the AI-VTCS offers three distinctive contributions relative to TAICS and other general AI-competency measures. First, it is specifically focused on the visualization domain, an aspect of teaching competency that these instruments do not delineate as a standalone construct. Second, rather than presenting dimensions as parallel components, it adopts a hierarchical structure (“theoretical dimensions—measurement dimensions—measurement items”) and empirically supports a second-order factor, thereby capturing the internal logic and interrelation among competency facets. Third, its Ethics and Safety dimension is tailored to the distinctive risks of visual data—such as the credibility of AI-generated visual information, algorithmic bias in visual content, and the pedagogical misuse of visualized outputs—rather than addressing ethics only in general terms. By constructing such a hierarchical framework, this study provides a theoretical basis for understanding the new connotations of teacher competency in the AI era. Second, the rigorously psychometrically validated AI-VTCS fills the gap in assessment tools within the current field of teacher AI literacy, offering a reliable and valid standardized measurement instrument (Allen and Kendeou, 2023). Importantly, the AI-VTCS does not replace TAICS but complements it, providing a domain-specific, hierarchically structured tool that can be used alongside general AI-competency measures for a more complete assessment of teachers’ AI readiness.

### 6.2 Practical implications

At the practical level, this study yields three implications, with specific use cases in teacher education. First, AI-VTCS can serve as a diagnostic assessment tool in pre-service and in-service teacher training programs. For example, training programs could administer the scale at the outset to profile each teacher’s strengths and weaknesses across the five dimensions, and then tailor instruction accordingly: teachers scoring low on the Design and Creation dimension could receive hands-on workshops in prompt engineering and the use of generative AI for creating effective visual representations (e.g., concept maps, data dashboards, and animations); those low on Awareness and Interpretation could be trained in critical visual data literacy, including techniques for detecting AI-generated misinformation and bias; teachers deficient in Decision-Making and Collaboration could engage in scenario-based modules on instructional decision-making and human–AI collaboration; those weak in Ethics and Safety could complete dedicated modules on AI data ethics, privacy protection, and algorithmic bias identification; and teachers low on Professional Development could be supported through teacher professional learning communities, structured teaching reflection protocols, and case-based discussions on AI visualization teaching. Second, the scale provides an operational tool for competency assessment amid educational digital transformation, translating abstract AI literacy requirements into measurable, intervenable indicators and advancing teacher professional development from experience-driven to data-driven approaches. Third, the theoretical framework and measurement instrument hold reference value for other countries and regions, and can support cross-regional and cross-cultural comparative research on teacher competency, providing a common measurement basis for global theoretical and practical dialogue in AI-era teacher education (Ng et al., 2023).

### 6.3 Limitations and future research

This study has four limitations that warrant further refinement in future research.

First, the limitation of sample generalizability. The sample was drawn from primary and secondary school teachers in western China, whose cultural backgrounds, educational resources, and levels of AI technology application may differ systematically from those in other regions. Therefore, prior to generalizing the scale to more developed regions in eastern China or to other countries, cross-cultural and cross-regional validation is warranted; future studies could examine the applicability and measurement invariance of the scale across broader geographical and cultural contexts, including more developed regions of eastern China and other countries, to enhance its generalizability.

Second, the methodological limitation of self-report measurement. As a self-report instrument, AI-VTCS may be susceptible to social desirability bias, as teachers may tend to overestimate their own AI visualization teaching competency. Future research could enhance response authenticity through procedural measures such as emphasizing anonymity and the exclusive use of data for academic research; meanwhile, objective assessment methods such as classroom observation and analysis of teaching artifacts could be incorporated to conduct multi-method triangulation of teachers’ actual AI visualization teaching competency.

Third, the absence of test–retest reliability. Test–retest reliability is a key indicator for assessing the temporal stability of a scale, which was not examined in this study. Future research could re-administer the scale to the same group of teachers after an appropriate interval to establish its test–retest reliability. In addition, future research could further establish the predictive validity of the AI-VTCS by linking teachers’ AI-empowered visualization teaching competency scores to student learning outcomes (e.g., academic achievement, learning motivation, and critical thinking), thereby verifying the practical educational value of this competency construct; intervention studies could also be conducted based on this scale to explore effective pathways and strategies for enhancing teachers’ AI visualization teaching competency.

Finally, the limitation of common method bias. Although Harman’s single-factor test suggested that common method bias was not a serious concern (the first factor accounted for 28.478% of the total variance, below the 40% threshold), the reliance on self-reported data means that common source variance cannot be entirely excluded. Future studies should consider marker variable techniques, multi-source ratings, or longitudinal designs to further reduce this risk.

## Data Availability

The raw data supporting the conclusions of this article will be made available by the authors, without undue reservation.

## Appendix

**Table A1:** The AI-empowered teacher visualization teaching competency scale.

Factor name	Item
Design & creation	1. I can use AI tools to transform abstract subject concepts into visual materials such as charts, images, or videos.
	2. I can fine-tune AI-generated visual content according to teaching objectives and student characteristics.
	3. I can integrate multimodal visual materials generated by AI into a coherent teaching presentation.
	4. I can creatively blend AI-generated visual materials with my own instructional designs.
	5. I can use AI tools to visualise students’ thinking processes (e.g., concept maps, mind maps).
	6. I can evaluate the suitability of different AI visualisation tools for specific teaching tasks.
	7. I can continuously iterate and improve my teaching presentation based on AI visual feedback.
	8. I can use AI tools to design differentiated visual learning resources appropriate for diverse proficiency levels.
Interpretation & awareness	9. I can accurately understand the meaning of each data indicator presented by AI learning analytics systems.
	10. I can identify key learning problems from AI-generated student data profiles.
	11. I can judge whether there are biases in data presentation in AI-generated teaching visualisation reports.
	12. I can translate instructional insights revealed by AI visualisation reports into specific teaching actions.
	13. I can synthesise students’ learning status from multiple AI visualisation data sources.
	14. I can recognise potential data limitations or uncertainties in AI-visualised information.
	15. I can connect AI-visualised information with my own subject-matter pedagogical knowledge to deepen teaching understanding.
Decision & collaboration	16. I can make final teaching decisions independently on the basis of AI-provided visual data.
	17. I can dynamically adjust classroom teaching strategies in real time based on data from AI learning analytics systems.
	18. I can recognise discrepancies between AI visualisation recommendations and my own teaching intuition and make reasoned choices.
	19. I can identify contextual factors that AI learning analytics systems may fail to capture and incorporate them into my decision-making.
	20. I can develop differentiated intervention plans based on AI visualisation data.
	21. When AI visualisation information is uncertain or incomplete, I can assess the potential risks and benefits of different teaching decisions.
Ethics & safety	22. I can identify student privacy information that may be involved in AI-generated visual data.
	23. I can ensure that the use of student data with AI visualisation tools complies with ethical standards.
	24. I can recognise algorithmic biases or discriminatory representations in AI visualisation outputs.
	25. I can judge whether AI-generated visual content may mislead particular student groups.
	26. I can guide students to view AI-generated visual information critically.
	27. I can weigh different options and make responsible decisions when facing ethical dilemmas in AI visual teaching.
Professional development	28. I can use AI visual feedback data to continuously reflect on and improve my teaching practice.
	29. I can set professional development goals based on teaching effectiveness evaluation results from AI learning analytics systems.
	30. I can share my experience of AI-empowered visual teaching with colleagues and facilitate mutual growth.
	31. I can proactively explore and experiment with new AI visual teaching strategies to enhance instructional effectiveness.
